# Liver Ischemia Reperfusion Injury, Enhanced by Trained Immunity, Is Attenuated in Caspase 1/Caspase 11 Double Gene Knockout Mice

**DOI:** 10.3390/pathogens9110879

**Published:** 2020-10-24

**Authors:** Alexander M. Fagenson, Keman Xu, Fatma Saaoud, Gayani Nanayakkara, Nirag C. Jhala, Lu Liu, Charles Drummer, Yu Sun, Kwan N. Lau, Antonio Di Carlo, Xiaohua Jiang, Hong Wang, Sunil S. Karhadkar, Xiaofeng Yang

**Affiliations:** 1 Department of Surgery, Division of Abdominal Organ Transplant, Lewis Katz School of Medicine, Temple University, 3401 N. Broad Street, Philadelphia, PA 19140, USA; Kwan.Lau@tuhs.temple.edu (K.N.L.); Antonio.dicarlo@tuhs.temple.edu (A.D.C.); Sunil.Karhadkar@tuhs.temple.edu (S.S.K.); 2 Centers for Cardiovascular Research, Inflammation, Translational and Clinical Lung Research, Metabolic Disease Research, Thrombosis Research, Lewis Katz School of Medicine, Temple University, Philadelphia, PA 19140, USA; keman.xu@temple.edu (K.X.); fatma.saaoud@temple.edu (F.S.); u6023761@utah.edu (G.N.); Charles.Drummer@temple.edu (C.D.); yusun379@temple.edu (Y.S.); xiaohua.jiang@temple.edu (X.J.); 3 Centers for Metabolic Disease Research, Cardiovascular Research and Thrombosis Research, Lewis Katz School of Medicine, Temple University, Philadelphia, PA 19140, USA; lulu.liu@temple.edu (L.L.); hong.wang@temple.edu (H.W.); 4 Eccles Institute of Human Genetics, University of Utah, Salt Lake City, UT 84112, USA; 5 Department of Pathology and Laboratory Medicine, Lewis Katz School of Medicine, Temple University, Philadelphia, PA 19140, USA; Nirag.Jhala@tuhs.temple.edu

**Keywords:** ischemia reperfusion injury, caspase 1, caspase 11, inflammasomes, trained immunity

## Abstract

Ischemia reperfusion injury (IRI) during liver transplantation increases morbidity and contributes to allograft dysfunction. There are no therapeutic strategies to mitigate IRI. We examined a novel hypothesis: caspase 1 and caspase 11 serve as danger-associated molecular pattern (DAMPs) sensors in IRI. By performing microarray analysis and using caspase 1/caspase 11 double-knockout (Casp DKO) mice, we show that the canonical and non-canonical inflammasome regulators are upregulated in mouse liver IRI. Ischemic pre (IPC)- and post-conditioning (IPO) induce upregulation of the canonical and non-canonical inflammasome regulators. Trained immunity (TI) regulators are upregulated in IPC and IPO. Furthermore, caspase 1 is activated during liver IRI, and Casp DKO attenuates liver IRI. Casp DKO maintained normal liver histology via decreased DNA damage. Finally, the decreased TUNEL assay-detected DNA damage is the underlying histopathological and molecular mechanisms of attenuated liver pyroptosis and IRI. In summary, liver IRI induces the upregulation of canonical and non-canonical inflammasomes and TI enzyme pathways. Casp DKO attenuate liver IRI. Development of novel therapeutics targeting caspase 1/caspase 11 and TI may help mitigate injury secondary to IRI. Our findings have provided novel insights on the roles of caspase 1, caspase 11, and inflammasome in sensing IRI derived DAMPs and TI-promoted IRI-induced liver injury.

## 1. Introduction

Ischemia reperfusion injury (IRI) is an unavoidable consequence during organ transplantation, hemorrhagic shock [1]/cardiogenic shock [2], myocardial infarction [3], and acute limb ischemia [4]. IRI following liver transplantation contributes to postoperative organ dysfunction, and increases the risk of acute and chronic rejection with subsequent graft failure [5]. Currently, no therapeutic options are available to mitigate IRI.

Allograft injury occurs during the cold ischemic [6] and warm [7] reperfusion phases. The underlying mechanism is a pro-inflammatory response mediated by the innate immune system [1,8]. Danger associated molecular pattern (DAMP) receptors including Toll-like receptors (TLRs), Nod-like receptors, and inflammasomes [9,10,11], have been reported in sensing IRI-derived sterile DAMPs, and bridging the DAMPs to inflammation-related tissue injury [12,13,14,15,16]. Activation of TLR4 on macrophages [17] triggers a cascade leading to inflammation and apoptosis/pyroptosis (inflammatory cell death) [18,19,20,21,22]. Liver sinusoidal endothelial cells are the first to become injured secondary to a microcirculatory disturbance that starts during the cold ischemic phase of organ preservation [6]. During the warm ischemic phase after reperfusion, a pro-inflammatory state occurs and apoptosis and pyroptosis are the mechanism by which cell death occurs [6,23,24]. Therefore, apoptosis and pyroptosis inhibition appears to be a therapeutic strategy for avoiding IRI. 

**Figure 1:**
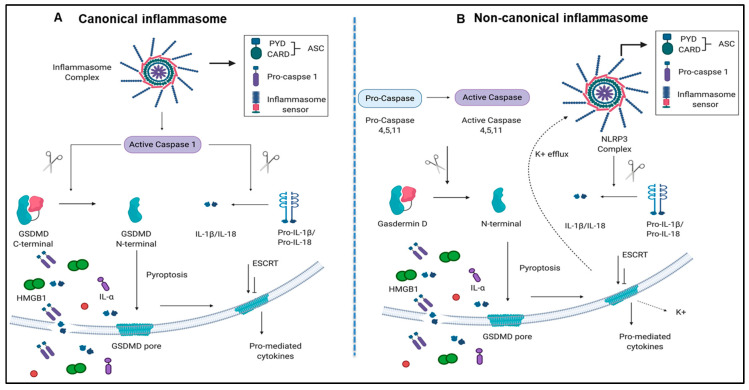
Both canonical and non-canonical inflammasome pathways play significant roles in inflammatory cell death (pyroptosis). (**A**) Canonical inflammasome complexes contain inflammasome sensors, which are used to recognize host-derived or pathogen derived danger signals. The structure of ASC contains a caspase recruitment domain that recruit pro-caspase 1. Caspase 1 can be activated within the inflammasome and cleaves GSDMD into C-terminal and N-terminal GSDMD fragments. N-terminal GSDMD alone can induce pyroptosis and form intrinsic pore on cell membrane while C-terminal GSDMD acts as a repressor that bind to N-terminal GSDMD to block its activity. In addition to GSDMD complexes cleavage, cytokines of pro-IL-1β and IL-18 can also be cleaved by caspase 1 and generate mature IL-1β and IL-18. When cells undergo pyroptosis, the GSDMD pores will release mature cytokines. (**B**) Non-canonical inflammasome pathway activates caspase 4, 5, (human), and 11 (mice) to cleave GSDMD in two steps. First, the potassium ions are released, leading to the activation of NLRP3 inflammasome and cytokine maturation. Second, pyroptosis is caused by GSDMD pores. Abbreviations: ASC, apoptosis-associated speck-like protein containing a caspase activation and recruitment domain (CARD); ESCRT: Endosomal sorting complexes required for transport (ESCRT) machinery is used to repair membrane damages that caused by the GSDMD pores; GSDMD, Gasdermin D; HMGB1, high mobility group box 1; IL-1β, interleukin-1β. The pictures were drawn with BioRender software.

Caspase family members, including caspases 2, 3, 6, 7, 8, 9, and 10, are involved in the classical pathway of apoptosis [25,26]. However, caspase 1 and caspase 4 (humans)/caspase 11(mice) are involved in pyroptosis [9,13,27]. Pyroptosis has been characterized into two pathways, a canonical caspase 1 pathway that generates interleukin 1 (IL-1β) and IL-18, and a non-canonical caspase 11/Gasdermin-D pathway that is involved in the assembly of N-terminal Gasdermin-D protein channel and the secretion of IL-1β and IL-18 [28,29] (Figure 1A,B). 

Inflammasomes are large protein complexes that act as a sensor for danger signals from pathogens and damaged cells, and activate caspase 1, which generate the cytokines IL-1β and IL-18. Once caspase 1 is activated and IL-1β secreted, IL-1β can circulate in the extracellular space leading to increased production of other pro-inflammatory cytokines and chemokines, which activate innate immune response and exacerbate inflammatory cascade [28,30,31,32]. Inflammasomes not only act as a sensor for danger signaling, but also can induce signaling amplification and activate a subsequent innate immune response and promote inflammation by secreting different cytokines and chemokines, therefore setting an increased threshold. Furthermore, several lines of evidence suggested that reactive oxygen species (ROS) can serve as a major inflammasome activator [13,33,34]. The ROS system can serve as an integrated sensor network related to the inflammasome/caspase system to trigger inflammation [34]. Inflammasomes have been shown to express in immune cells, including monocytes, macrophages, neutrophils, T cells, natural killer (NK) cells, and dendritic cells. There is increasing evidence that inflammasomes exist and are functionally active in other non-immune cells, including hepatocytes [35,36]. Dysregulation of inflammasome plays a significant role in different liver diseases, including liver injury [37,38], liver fibrosis and cirrhosis [39,40], and alcoholic and non-alcoholic fatty liver diseases [35,41]. Caspase 1/inflammasome serve as a danger signal involved in IRI [10,12,14,15,42,43,44]. Interestingly, rat liver transplantation in the presence of a pan-caspase inhibitor showed improved liver function post-transplantation [43,45,46]. 

Inflammasome adaptor protein ASC (apoptosis-associated speck-like protein containing a CARD domain) deficiency, IL-1β blocking antibody injection [43], injection of Serp 2 (a virus-derived serine protease and pan-caspase inhibitor) [47], and caspase 1/caspase 4 substrate Gasdermin D deficiency [48] lead to protection against liver IRI [49]. Caspase 1 deficient mice are less susceptible to acetaminophen-induced liver injury [50]. Recently, it has been reported that caspase 1 knockout mice are de facto caspase 1 and caspase 4 (humans)/caspase 11 (mice) double knockout mice [51]. The situation resulted from the dysfunctional nature of the naturally occurring 129 caspase 11 allele whereas these caspase 1 knockout mice were produced in embryonic stem cells on a 129S2 background [25]. Thus, the Casp DKO mice are ideal for determining whether the canonical and non-canonical inflammasome pathways play critical roles in the liver IRI pathogenesis. However, the effects of Casp DKO [51] in liver IRI has not been studied. 

The activation of the innate immune system results in enhanced responsiveness to subsequent triggers, which is termed trained immunity (TI) [52,53]. Pro-atherogenic lysophosphatidylcholine (LPC) upregulates trained immunity pathways (TIPs) in human aortic endothelial cells (HAECs) [54], however it is unknown whether liver IRI is enhanced by ischemic pre (IPC) and post-conditioning (IPO) through upregulation of TIPs. IPC, IPO, and IPC+IPO had pronounced effects on the expression levels of a large number of genes during early reperfusion [55]. However, whether reperfusion, IPC, IPO, and IPC+IPO upregulate inflammasome regulators and enhance ischemia-induced injury via TI-mediated mechanisms still unknown.

Our central hypotheses are liver IRI induces transcriptomic changes of canonical and non-canonical inflammasome regulators, which may be promoted by TIPs, and deficiency of caspase 1/caspase 11 will decrease liver ischemic damage. 

## 2. Results

### 2.1. Two Canonical and Two Non-Canonical Inflammasome Regulators Are Upregulated in Mouse Liver IRI

The canonical inflammasome and caspase 1 pathway play a critical role in sensing liver IRI-derived DAMPs and activate caspase 1 to generate mature pro-inflammatory cytokines IL-1β and IL-18 [13] (Figure 1A). Additionally, non-canonical inflammasome and caspase 4/caspase 11 are responsible for the formation of protein channel/pores on the plasma membrane to release mature IL-1β and IL-18 and pyroptosis [56] (Figure 1B). However, whether liver IRI induces activation of inflammasome pathways remains unknown.

**Figure 2:**
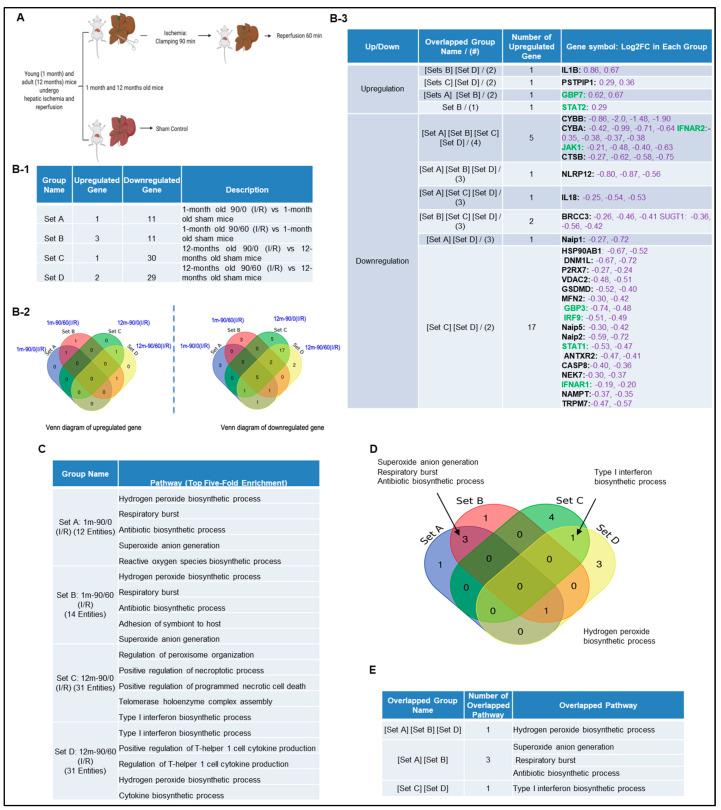
A mouse liver IR microarray dataset from the NIH-NCBI-GeoDatasets database (https://www.ncbi.nlm.nih.gov/geo/). (**A**) Schematic presentation of experimental design for the microarray analysis [61] GSE10652/10657. Total RNA of young (1 month) and adult (12 to 14 months) mice, which underwent sham surgery or partial hepatic ischemia for 90 min. The 90 min of ischemia group mice followed by 60 min reperfusion were analyzed by Affymetrix microarray. (**B-1**) The detailed description of group classification and a summary of pyroptosis gene changes (up-/downregulation) in each group. (**B-2**) The Venn-diagram of up and downregulated genes that are shown in (**B-1**). (**B-3**) The details of overlapped genes groups, gene names, and fold changes (Log2FC) of each gene. To Note: These up and downregulated genes were significantly changed compared to the sham group (*p* value < 0.05). Non-canonical genes were marked in green. (**C**) We used GENEONTOLOGY to analyze the four groups of genes in (**B-1**) and found the top five-fold enrichment pathways that are related to each of the group entities. The gene list of each group is listed in Supplementary Tables S1–S4. (**D**) The Venn-diagram of the four groups pathways. (**E**) The overlapped pathway among the four groups. The pathway of the hydrogen peroxide biosynthetic process was held in three groups except in Set C.

**Figure 3:**
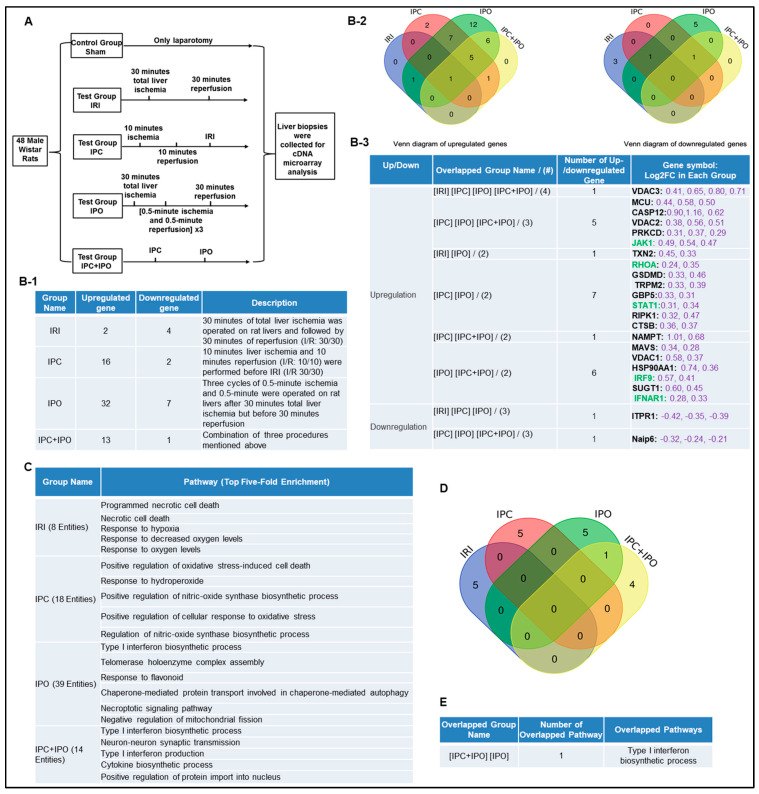
Ischemic pre (IPC)- and post-conditioning (IPO) induce upregulation of canonical and non-canonical inflammasome regulators more than liver IRI. A database mining work (GSE24430) of rat liver ischemia-reperfusion with the effects of ischemic pre- (IPC) and post-conditioning (IPO). (**A**) Schematic presentation of experimental design for the microarray analysis [55]. IPC (10 min ischemia/10 min reperfusion before ischemia for 30 min/reperfusion for 30 min) and IPO [(0.5 min ischemia/0.5 min reperfusion) × 3) after 30 min ischemia]. (**B-1**) The detailed description of group classification and a summary of pyroptosis gene changes (up-/downregulation) in each group. (**B-2**) The Venn-diagram of up and downregulated genes shown in (**B-1**). (**B-3**) The details of overlapped gene groups, gene names, and fold changes (Log2FC) of each gene. To Note: These up and downregulated genes were significantly changed compared to the sham group (*p* value < 0.05). Non-canonical genes were marked in green. (**C**) We used the GENEONTOLOGY website to analyze the four groups (IRI, IPC, IPO, IPC + IPO) of genes in B-1 and found the top five-fold enrichment pathways that are related to each of the group entities. The gene list of each group were listed in Supplementary Table S5. (**D**) The Venn diagram of the four group pathways. (**E**) The overlapped pathway among the four groups. Only one pathway—the type I interferon biosynthetic process—was held by both the IPC and IPC+IPO groups. The other pathways were exclusive in each group.

Next, we hypothesized that canonical and non-canonical inflammasome pathway regulators are upregulated in liver IRI. To test this, we used the NIH-NCBI GeoDatasets database and our pioneered methods in database mining [57,58,59,60] to analyze the expressions of 96 canonical/non-canonical inflammasome/pyroptosis genes in a microarray dataset performed on hepatic ischemia/reperfusion mouse models [61] (Figure 2A). The list of 96 inflammasome pathways and pyroptosis genes were newly collected in the Kyoto Encyclopedia of Genes and Genomes (https://www.genome.jp/kegg/), thus, the results generated from our database mining were novel. The datasets were divided into four sets (A, B, C, and D): 12 young (1 month) and 12 adult (12 months) mice experienced 90 min ischemia followed either with or without 60 min reperfusion (Figure 2B-1). Of note, this original paper was to study age-dependent response to IRI, but our study aims to determine whether liver IRI induces activation of inflammasome and caspase 1 pathway. Our results showed that one gene was upregulated, and 11 genes were downregulated in the set A. Three genes were upregulated, and 11 genes were downregulated in the set B. One gene was upregulated, and 30 genes were downregulated in the set C. In addition, two genes were upregulated, and 29 genes were downregulated in the set D. Furthermore, we used the Venn-diagram analysis to identify the unique upregulated genes in each dataset (Figure 2B-2). Four inflammasome regulators were significantly upregulated including IL-1β (sets B and D), proline-serine-threonine phosphatase interacting protein-1(PSTPIP1) (sets C and D), guanylate binding protein-7 (GBP7) (sets A and B), and signal transducer and activator of transcription-2 (STAT2) (set B) (Figure 2B-3). These results have demonstrated that the IL-1β upregulation is induced by reperfusion [13] since it was upregulated in the sets B and D. PSTPIP1 was induced when aging liver (12 month-old mouse livers), suggesting that inflammasome pathways are suppressed since PSTPIP1 inhibits inflammasome activation [62]. Furthermore, GBP7 may be involved in host defense against intracellular bacteria and parasites since it has been reported that two GBP7 homologous human GBP1 is functional in this front [63]. GBP5 plays a role in inflammasome assembly [64]; and GTPase activity of Gbp4 is indispensable for inflammasome activation and *Salmonella Typhimurium* clearance [65]. Finally, liver IRI induces downregulation more than upregulation of inflammasome regulators, suggesting that liver IRI is selective in choosing inflammasome regulators. We further examined a hypothesis that several shared signaling pathways underlined the four groups of transcriptomic changes related to liver IRI in young and old mice using the GENEONTOLOGY website (http://geneontology.org/). Five top pathways were identified based on the gene changes in each group (Figure 2C). Additionally, we used Venn-diagram analysis to identify the shared and unique pathways in each group (Figure 2D). We showed that three pathways were unique in liver IRI in young mice including superoxide anion generation, respiratory burst and antibiotic biosynthetic process. The type-1 interferon biosynthetic process was unique in liver IRI in old mice presumably related to cell senescence [66], and the hydrogen peroxide biosynthetic process was shared in liver IRI in both young and old mice (Figure 2E). These results conclude that the older mice are different from younger mice; however, in the future we need to set up experiments to further study this variation in detail. 

### 2.2. Ischemic Pre (IPC)- and Post-Conditioning (IPO) Induce Upregulation of Canonical and Non-Canonical Inflammasome Regulators, More than Liver IRI

IPC, IPO, and IPC+IPO had pronounced effects on the expression levels of a large number of genes during early reperfusion [55]. We hypothesized that IPC and IPO (Figure 3A) have pronounced effects in upregulating inflammasome regulators. To test this, we examined another dataset to analyze the expression of 96 canonical and non-canonical inflammasome pathway regulators. We showed that liver ischemia (30 min)/reperfusion (30 min) upregulated two genes and downregulated four genes. IPC upregulated 16 genes and downregulated two genes, IPO upregulated 32 genes and downregulated seven genes. However, IPC+IPO upregulated 13 genes and downregulated one gene (Figure 3B-1). Then, we used the Venn-diagram analysis to identify the unique upregulated genes in each dataset (Figure 3B-2,B-3). These results have demonstrated that the IPC and IPO significantly increased more gene upregulation than IRI alone, and IPC+IPO resulted in less gene upregulation than that of IPC and IPO alone but more than that of IRI. In addition, we showed that IPO induced the strongest responses in upregulating inflammasome regulators among the four groups. These results suggest that two groups IPC and IPO have more enhanced effects than IRI, induce more gene upregulation, and have no synergistic effects in comparison to IPC and IPO alone. We further examined a hypothesis that several shared signaling pathways underline the transcriptomic changes in these four groups (Figure 3C). We showed that IRI induced two high fold enrichment pathways such as programmed necrotic cell-death, and necrotic cell-death. IPC induced the top five pathways with the concentration of oxidative stress responses and IPO induced the top five pathways with the concentration of type-I interferon biosynthetic process, telomerase, chaperone, and mitochondrial fission responses. In addition, there is one pathway, type-I interferon biosynthetic process, shared by IPC+IPO and IPO alone (Figure 3D,E). These results have demonstrated that IPC and IPO have more pathways in cellular responses to oxidative stress than IRI. Of note, 2–3% of 96 inflammasome regulators upregulated in mouse IRI in Section 1 are similar to that (2.1%) of rat liver IRI, suggesting that rodent liver IRI pathways and mechanisms are highly conserved. 

### 2.3. Trained Immunity Regulators Are Upregulated in Pre-Conditioning and Post-Conditioning Much More than Liver IRI

**Figure 4:**
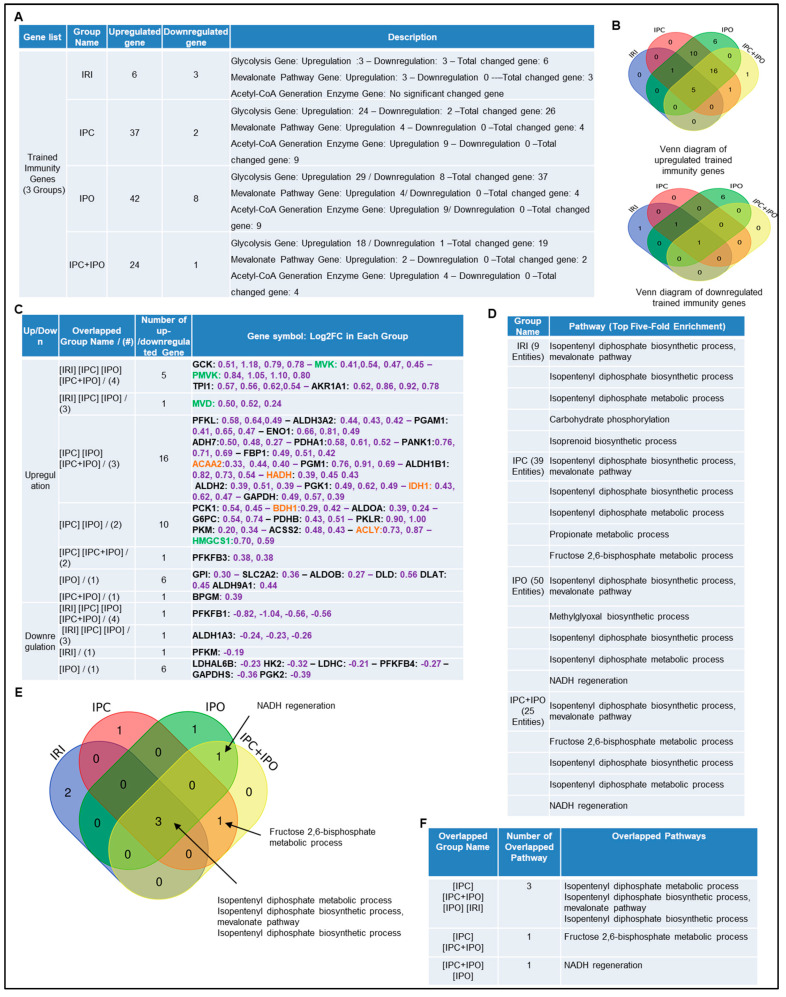
Ischemic pre (IPC)- and post-conditioning (IPO) induce upregulation of trained immunity regulators more than liver IRI. A database mining work (GSE24430) of rat liver ischemia-reperfusion with trained immunity pathway (TIP) enzymes. (**A**) The detailed description of group classification and a summary of TIP enzyme gene changes (up-/downregulation) in each group. (**B**) The Venn-diagram results of up- or downregulation genes that are shown in A. (**C**) The details of overlapped gene groups, gene names, and fold changes (Log2FC) of each gene. *p* value < 0.05. Glycolysis, acetyl-CoA, mevalonate pathway enzymes were marked in black, orange, and green, respectively. (**D**) We used the GENEONTOLOGY website (http://geneontology.org/) to analyze four groups (IRI, IPC, IPO, IPC+IPO) of TIP genes in C and found the top five-fold enrichment pathways that are related to each of the group entities. (**E**) The Venn-diagram of four group pathways. (**F**) The overlapped pathway among the four groups mentioned above. The total gene list of each group can be found in Supplementary Table S6.

We hypothesized that enhancements of liver IRI by IPC and IPO are associated with increased TIPs expressions. To test this, we examined the expression changes of 102 TIP enzymes including 71 glycolysis enzymes, 7 mevalonate pathway enzymes, and 24 acetyl-CoA generation enzymes [54], in the four groups of microarrays. We demonstrated that IRI upregulated six TIP enzyme genes (three glycolysis genes, three mevalonate genes and no acetyl-CoA genes) and downregulated three TIP enzyme genes. IPC upregulated 37 TIP enzymes genes (24 glycolysis genes, four mevalonate genes, and nine acetyl-CoA genes) and downregulated two TIP enzyme genes. IPO upregulated 42 TIP enzyme genes (29 glycolysis genes, four mevalonate genes, and nine acetyl-CoA genes) and down regulated eight TIP enzymes genes. Furthermore, IPC+IPO upregulated 24 TIP enzymes genes (18 glycolysis genes, two mevalonate genes, and four acetyl-CoA genes) and downregulated one TIP enzyme gene (Figure 4A). Then, we used the Venn diagram to examine the up and downregulated genes (Figure 4B,C). These highly innovative findings have demonstrated that the IPC and IPO induce much more TIP enzymes than liver IRI. TIPs may play significant roles for enhancing inflammasome gene upregulations [67]. IPC and IPO induce significant upregulation of acetyl-CoA synthesis enzymes but IRI does not. However, IPC and IPO significantly induce glycolysis enzymes. We also used the GENEOTOLOGY website to analyze the TIP enzyme genes in these four groups and found the top five-fold enrichment pathways that are related to each group (Figure 4D). These results demonstrated that all the four groups induced the top five pathways. There are three pathways shared by the four groups. However, there is only one pathway shared by IPC+IPO and IPC alone and one pathway shared by IPC + IPO and IPO alone (Figure 4E,F). Our results have demonstrated for the first time that IPC, IPO and reperfusion activate TIP enzymes, enhance TI and amplify upregulation of inflammasome regulators and IRI.

### 2.4. Caspase 1 Is Activated during Liver IRI, Suggesting that Post-translational Inflammasome Protein Complex Assembly Can also Be a Mechanism Underlying Liver IRI in Addition to Transcriptional Upregulation of Inflammasome Regulators

**Figure 5:**
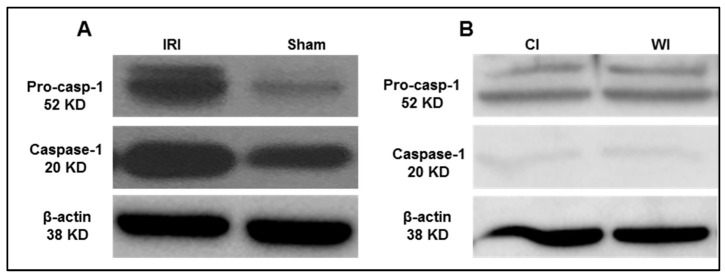
Caspase-1 activation is increased after liver ischemia-reperfusion. (**A**) Wild-type C57BL/6 (WT) mice were subjected to 45 min of total ischemia followed by 2 h of reperfusion. Sham WT mice were used as a control and were subjected to the same surgical conditions but without vascular occlusion. (**B**) WT mice were subjected to 24 h cold ischemia (CI) alone and 45 min warm ischemia (WI) alone. n = 6 mice per group.

Based on the results from the transcriptomic database mining analysis, we generated a hypothesis that caspase 1 is activated in mouse liver I/R. To test this hypothesis, we first performed the liver IRI in wild-type C57BL/6 (WT) mice. An increased expression of activated caspase 1 was observed after 45 min of total liver ischemia followed by two hours of reperfusion (Figure 5A). However, in the first control experiment livers were procured without ischemia and stored in the cold at the University of Wisconsin solution for 24 h, we did not observe activated caspase 1 expression (Figure 5B). Furthermore, as the second control experiment, interruption of blood flow to induce ischemia only without subsequent reperfusion did not lead to activation of caspase 1, which was correlated well with our earlier findings in the data mining analysis. Reperfusion after ischemia has enhanced tissue injury responses compared to that of ischemia alone potentially due to upregulation of TIP enzymes and innate immune memory function. Taken together, our results have demonstrated that caspase 1 is activated during liver IRI.

### 2.5. Caspase 1/Caspase 11 Double Knockouts Attenuate Liver IRI, Suggesting that Upregulated Inflammasome Regulators and Activated Caspase 1 Play a Causative Effect for Promoting Liver IRI

**Figure 6:**
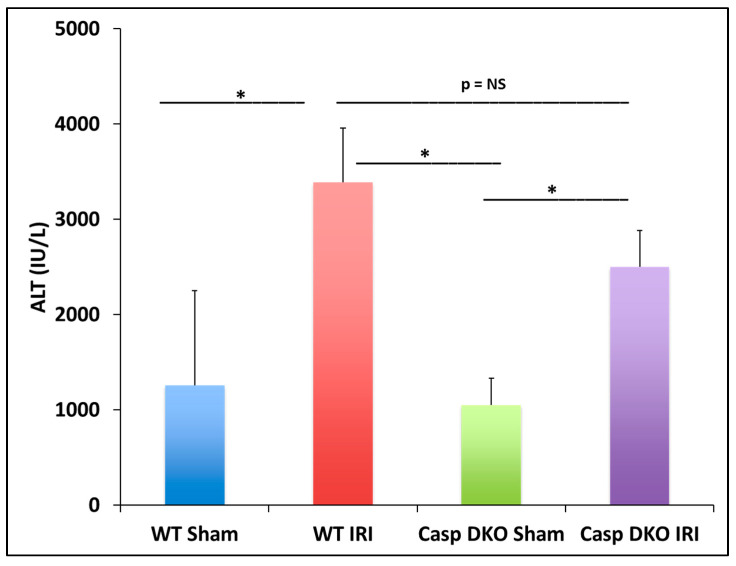
Casp double-knockout (DKO) mice reduced hepatocellular function as measured by serum alanine aminotransferase (ALT). WT and Casp DKO mice had a significant increase in ALT levels following IR. Decreased levels of ALT were observed in Casp DKO mice compared to WT mice. Means and standard deviations are shown. n = 6 per group. * *p* < 0.05.

In the previous section, we found some caspase 1-dependent canonical inflammasome and caspase 11-dependent non-canonical inflammasome regulators are upregulated in liver IRI, IPC, IPO, and IPC+IPO. We hypothesized that liver IRI is decreased in Casp DKO mice. With respect to hepatocyte injury, WT and Casp DKO mouse livers both showed significant elevations in alanine aminotransferase (ALT) after IRI compared to their respective sham controls (Figure 6), suggesting that the liver IRI resulted in liver injury. Furthermore, Casp DKO mice showed a reduction in ALT when compared to WT following IRI but did not reach statistical significance (2500 U/L vs. 3390 U/L) (Figure 6).

### 2.6. Caspase 1/Caspase 11 Double Knockouts Decrease DNA Damage, Which Is the Underlying Histopathological and Molecular Mechanisms of Attenuated Liver Apoptosis/Pyroptosis and IRI

**Figure 7:**
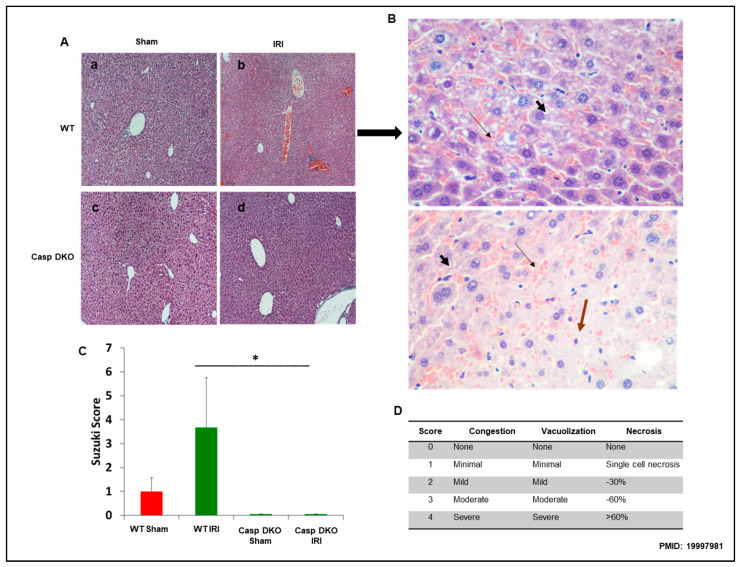
Casp DKO mice retain normal liver histology and decreased cellular injury after IRI. Casp DKO and WT mice were both subjected to 45 min of total ischemia followed by 2 h of reperfusion. (**A**) Representative H&E staining of liver slides. Sham WT (a) and Casp DKO (c) both exhibited normal liver histology. WT mice (b) demonstrated significant (*p* < 0.05) injury as measured by Suzuki grading classification after IR, whereas Casp DKO mice retained a normal liver architecture following IR (d). (**B**) WT mouse liver with areas of congestion (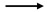) as highlighted by thin arrow and microvesicular steatosis in hepatocytes by thick short arrow (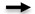) with dead (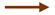) or degenerating hepatocytes extending from Zone 2 to Zone 3. (Stain: Hematoxylin and Eosin; Magnification: ×40). (**C**) Represents quantification by Suzuki classification grading scale. (**D**) Portrays the Suzuki classification grading scale. Means and standard deviations are shown. n = 6 per group. * *p* < 0.05.

To determine molecular mechanisms underlying decreased ALT elevation in Casp DKO mice, we performed histological examinations in mouse livers. Casp DKO mice exhibited normal liver architecture after IRI when compared to WT mice undergoing IRI (Figure 7A). Figure 7B showed areas of congestion, microvesicular steatosis in hepatocytes, and degenerating hepatocytes in WT IRI mice (Suzuki score = 3.6). Casp DKO IRI mice showed significantly less congestion, vacuolization and necrosis (Suzuki score = 0) (Figure 7C) [68]. 

**Figure 8:**
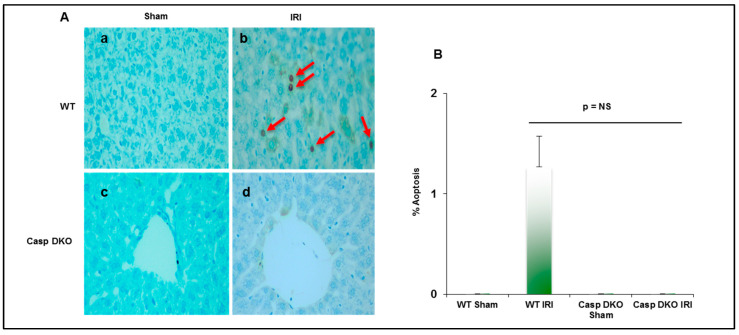
Casp DKO mice exhibit decreased cell death following ischemia-reperfusion. (**A**) Casp DKO and WT mice were both subjected to IRI and cell death was measured by the TUNEL assay. Casp DKO (a) and WT Sham (b) control groups had no TUNEL+ apoptotic/pyroptotic cells present. However, WT mice undergoing IR had increased TUNEL+ apoptotic/pyroptotic cells while Casp DKO mice had no TUNEL+ cells (d). (Magnification ×40). Red arrows indicated TUNEL+ apoptotic cells. (**B**) Quantification of TUNEL assay. Mean and standard deviation are shown. n = 6 per group.

Since both pyroptosis and apoptosis are programmed cell death and share several features including DNA damage detected by TUNEL assay [69], we performed TUNEL assay to detect DNA damage. Casp DKO mice had minimal pyroptosis and apoptosis following IRI, whereas WT mice had increased pyroptosis and apoptosis (0 versus 16 TUNEL^+^ cells per HPF) (Figure 8A,B).

## 3. Discussion

In this study, we wanted to determine if liver ischemia, IPC and IPO upregulate the canonical and non-canonical inflammasomes. In addition, we sought to investigate whether liver IRI, IPC and IPO increased upregulations of inflammasome are associated with upregulation of TI, whether liver IRI activates caspase-1, and if Casp DKO attenuates liver IRI. Reactive oxygen species system can serve as an integrated sensor network to sense different stimuli and connected to inflammasome activation and TI [34]. Caspase 1 serves as a danger signal for inflammation and IRI [70]. Apoptosis-associated speck like protein contain a CARD domain (ASC) is essential for generation of the inflammasome and inducing caspase 1 activation [71]. ASC-deficiency in a partial liver ischemia showed an inhibition in the caspase 1/IL-1β signaling and protection against liver IRI [43]. However, whether liver ischemia induces hypoxia-derived DAMPs and canonical and non-canonical inflammasome regulator upregulation is unknown. By using database-mining analysis, we demonstrated that two canonical and two non-canonical inflammasome regulators are upregulated in mouse liver IRI. IPC and IPO induce upregulation of canonical and non-canonical inflammasome regulators, more than liver IRI. TI regulators are upregulated in IPC and IPO much more than liver IRI. Furthermore, we used western blot analysis and found that caspase 1 is activated during liver IRI, suggesting that inflammasome protein complex assembly can also be a mechanism underlying liver IRI in addition to transcriptional upregulation of inflammasome regulators. Furthermore, by using our Casp DKO mouse model and examining liver function and histologic assessment we have shown that Casp DKO attenuates liver IRI, suggesting that upregulated inflammasome regulators and activated caspase 1 play a causative effect for promoting liver IRI.

**Figure 9:**
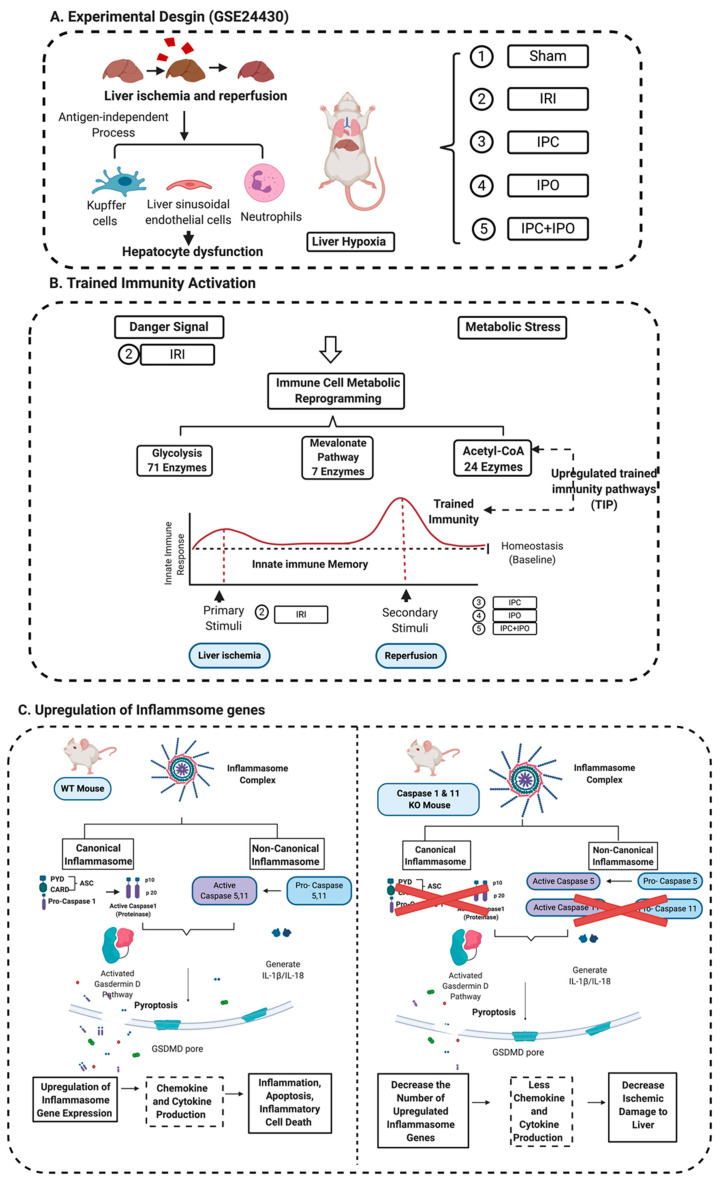
A novel working model. (**A**) Liver ischemia and reperfusion injuries are majorly caused by the formation of ROS that are released from Kupffer cells, the swelling of liver sinusoidal endothelial cells in the early stage of reperfusion, and the inflammation of neutrophil infiltration in the late stage leading to hepatocyte dysfunction. This figure showed an experimental design from GSE 24430 and through the database mining, we found the liver hypoxia or IRI act as inducers that can be sensed by inflammasome pathways. (**B**) Inflammasomes not only act as a sensor for danger signaling, but also can cause signaling amplification and activate a subsequent innate immune response, so called trained immunity activation. Based on the database mining, we believed that IPC, IPO, and reperfusion play a role in secondary stimuli to activate TIP enzymes and amplify upregulation of inflammasome regulators and IRI. (**C**) The combination of database mining results and animal study, we found liver IRI promoted by trained immunity and attenuated in Casp DKO mice.

Caspase 1 generates IL-1β and IL-18 in the canonical inflammasome pathway of pyroptosis [72]. Caspase 11 is the mediator for the non-canonical inflammasome pathway. Inflammasome activation leads to activation of caspase 1 and caspase 11, which are responsible for cleaving the N-terminus of Gasdermin D, which forms protein pore/channel for releasing IL-1β and IL-18 and pyroptosis [56]. Those released cytokines and chemokines can enhance immune response and exacerbate inflammation [30]. Caspase inhibition protects against ischemic injury in the brain [73], heart [74], lung [75] and liver [45,46,76]. To our knowledge, this is the first report of a Casp DKO murine model [25,51] to be used in studying hepatic IRI. Cell death rates in liver were lower in Casp DKO mice than WT mice. Furthermore, hepatocyte injury was reduced in the Casp DKO mice. These findings will potentiate identification of novel therapeutics to mitigate hepatic IRI in a liver transplantation setting by inhibition of caspase 1 (Figure 9). 

Previously, we discovered that inflammasomes are differentially expressed in various tissues [58]. Caspase 1 recognizes extended cleavage sites in its natural substrates [14], Casp DKO mice inhibits carotid neointimal hyperplasia [77], Casp DKO mouse in murine hind-limb ischemia model shows improved blood flow and angiogenesis [12], Casp DKO in apolipoprotein-E KO background decreases atherosclerosis [16], Casp DKO inhibits cardiovascular risk factor hyperhomocysteinemia-induced pyrop-apoptosis in endothelial cells [78], and Casp DKO improves progenitor cell vessel repair in ischemic heart [15]. Of note, our previous reports suggested the roles of caspase 1, which are actually attributed to both caspase 1/caspase 11 due to the Casp DKO mice we used [25,51]. We also reported that novel extracellular caspase 1 and inflammasomes propagate inflammation [10] and caspase 1 regulates gene expression via pathways independent of IL-1β, IL-18 and sirtuin 1 [11].

The activation of the innate immune system results in enhanced responsiveness to subsequent triggers, which is termed TI [52,53]. We recently reported that LPC upregulates TIPs in HAECs [54]. An important question remains whether liver IRI is enhanced by IPC and IPO through upregulating TIPs. Our findings showed that TI regulators are upregulated in IPC and IPO. The results provide novel insight on molecular mechanisms underlying reperfusion, IPC, IPO, and IPC+IPO enhancement of ischemia-induced liver injury and new TI-based therapeutics [79,80] for liver IRI as well as other tissue IRIs including myocardial infarction/reperfusion-induced, ischemic stroke/reperfusion-, hind-limb ischemia-induced injuries [12,15]. 

Mitigation of ischemic liver injury through pan-caspase inhibition has been shown in multiple cold and warm ischemic models. A pan-caspase inhibitor (IDN-1965) showed to prevent apoptosis of sinusoidal endothelial cells and improved survival after rat liver transplantation [6]. However, the inhibitor was required to be present in the storage solution as well as injected to the donor and recipient. A second-generation inhibitor (IDN-6556) that only needed to be present in the storage solution showed the same survival benefit [46]. An ex vivo rat liver perfusion model showed that livers pumped in the presence of IDN-6556 showed a reduction in liver injury [45]. Furthermore, an improved survival in rats undergoing liver transplantation was found when the liver was stored in the presence of the inhibitor. These studies show that the non-selective pan-caspase inhibitors could be used to inhibit apoptosis after liver transplantation. Our data demonstrate that the Casp DKO mitigates liver IRI and can be used as a selective target. Furthermore, a pan-caspase inhibitor such as IDN-6556 can inhibit multiple pathways leading to alternate damage.

A phase 2 human clinical trial with IDN-6556 (Emricisan) in liver transplantation patients [81] showed that groups having IDN-6556 in the flush and storage solution had the most protective effect against IRI. Interestingly, when the inhibitor was given for 24 h post-transplantation the positive effects were overturned. This may be related to the accumulation of neutrophils in the allograft. As neutrophil turnover is an apoptosis dependent manner, the presence of the inhibitor may potentiate neutrophils in the allograft and cause them to linger contributing to the postoperative inflammatory state.

There are several limitations of this study including: murine models are not always representative of the physiology present in humans. Our model is of liver ischemia/reperfusion through total liver ischemia, and does not represent the complete cycle ischemia/reperfusion occurring during liver transplantation. Additionally, our data represent short-term outcomes related to liver surgery. Future work involves using the Casp DKO mice to study renal IRI, as well as the administration of a caspase 1 specific inhibitor to WT mice prior to IRI. Furthermore, future work using PCR analysis is needed to confirm the up-, and down- regulated genes from the database mining analysis using WT and Casp DKO IRI tissue samples.

In summary, liver IRI, IPC, IPO and IPC+IPO resulted in increased expression of canonical and non-canonical inflammasomes as well as TIPs enzymes. This is the first study to demonstrate that Casp DKO mice retain normal liver architecture, decreased cell death after IRI, and attenuated liver IRI. Thus, we argue that caspase 1/caspase 11 and TIPs are novel therapeutic targets for the reduction of IRI.

## 4. Materials and Methods

### 4.1. Animals

Wild-type C57BL/6 (WT) mice and Casp DKO mice [25] were held in the Temple University Lewis Katz School of Medicine animal facility under pathogen-free conditions. All proposed experiments were approved by the Institutional Animal Care Use Committee.

### 4.2. Animal Surgeries 

Sex-matched, 10–12 week-old WT and Casp DKO mice were anesthetized with sevoflurane. A midline laparotomy was performed and mice were injected with 100U of heparin systematically into the inferior vena cava (IVC). After five minutes, an atraumatic vascular clip (Edwards Lifesciences) was applied to the portal vein and hepatic artery interrupting blood flow for 45 min. Then, the clip was removed, and the mice were returned to their cages for 2 h of reperfusion. Following reperfusion, blood was collected from the IVC and livers were flushed with cold University of Wisconsin solution (Bridge to Life). 

### 4.3. Western Blots

Total protein was extracted from liver tissue. A total of 25 μg of protein was loaded on a 12.5% SDS-PAGE and transferred to a nitrocellulose membrane. The membranes were then probed for caspase 1 with anti-caspase 1 antibody (cell signaling) along with a house keeping β-actin control.

### 4.4. Hepatocellular Damage Assay

Blood was collected from the IVC following 2 h of reperfusion. Plasma was isolated by blood centrifugation for 30 min at 4 °C. Serum alanine aminotransferase (ALT) was measured using Thermo Scientific™ ALT/GPT Reagent (Fisher Scientific).

### 4.5. Liver Histology 

Livers were fixed for 24 h in 4% paraformaldehyde at 4 °C and stored in 70% ethanol, then de-paraffinized and sectioned into 4 μm sections. Haemotoxylin/eosin staining performed and liver injury was graded by a liver pathologist using the Suzuki classification [68,82].

### 4.6. Cell Death Assay

Terminal deoxynucleotidyl transferase dUTP nick end labeling (TUNEL) assay was performed on paraffin-embedded slides according to manufacturer’s instructions (Abcam).

### 4.7. Expression Profile of Canonical and Non-Canonical Inflammasome Genes in Mouse Liver with IRI

Microarray datasets were collected from the National Institutes of Health (NIH)-National Center for Biotechnology Information (NCBI)-Gene Expression Omnibus (GEO) databases (https://www.ncbi.nlm.nih.gov/gds) and analyzed with an online software GEO2R (https://www.ncbi.nlm.nih.gov/geo/geo2r/). The detailed information of the GEO datasets was shown in Figure 2 and Figure 3 and Supplementary Tables using our pioneered big data mining strategy [57,58,59,60]. 

### 4.8. Statistics

Experiments were performed at least three times and differences are expressed as means and standard deviations. Statistical comparisons between experimental groups were compared by paired Student’s *t* test or one-way analysis of variance. A *p* < 0.05 was considered statistically significant.

## Supplementary Materials

The following are available online at https://www.mdpi.com/2076-0817/9/11/879/s1, Table S1: 41 unique genes were significant changed (*p* value < 0.05 and Log2 fold change was shown in the last column) in young and adult mice of 30, 60, 90 minutes ischemia [Ischemia/Reperfusion (I/R): 30/0, 60/0, 90/0] verse sham control in the dataset of GSE10652. Table S2: 36 unique genes were significant changed (*p* value < 0.05 and Log2 fold change was shown in the last column) in young and adult mice of 90 minutes ischemia and 60 minutes reperfusion (I/R: 90/60) groups of the dataset GSE 10657. Table S3: Summary table of 41 unique significantly downregulated (*p* value < 0.05) genes (Canonical_34 vs non-canonical_7) in young and adult mice after hepatic ischemia 90 minutes and reperfusion 0, and 60 minutes, respectively. Table S4: Summary table of seven unique significantly upregulated (*p* value < 0.05) genes (Canonical_5 vs non-canonical_2) in young and adult mice after hepatic ischemia 90 minutes and reperfusion with 0, and 60 minutes, respectively. Table S5: 45 unique pyroptosis genes were significant changed (*p* value < 0.05 and fold change (Log2FC) was shown in the last column) in 48 male rats that were subjected to liver ischemia-reperfusion (IRI), ischemic pre- (IPC), post-conditioning (IPO), and IPC+IPO in the dataset of GSE24430. Table S6: 49 unique trained immunity pathway enzymes were significant changed (*p* value < 0.05 and fold change (Log2FC) was shown in the last column) in 48 male rats that were subjected to liver ischemia-reperfusion (IRI), ischemic pre- (IPC), post-conditioning (IPO), and IPC+IPO in the dataset of GSE24430.
