# Supplementary material for: Liver Ischemia Reperfusion Injury, Enhanced by Trained Immunity, Is Attenuated in Caspase 1/Caspase 11 Double Gene Knockout Mice
Source: Pathogens. 2020 Oct 24;9(11):879. doi: 10.3390/pathogens9110879 (PMC7692674; doi:10.3390/pathogens9110879)
Supplement: Supplementary file 1 [file pathogens-09-00879-s001.pdf]

Supplementary materials

Table S1. 41 unique genes were significant changed (P value < 0.05 and Log2 fold change was shown in the last column) in young and adult mice of 30, 60, 90 minutes ischemia [Ischemia/Reperfusion (I/R): 30/0, 60/0, 90/0] verse sham control in the dataset of GSE10652. * Canonical genes (33) are shown in black and non-canonical genes (8) are shown in bold green. 96 inflammasome pathways/pyroptosis genes were found in the Kyoto Encyclopedia of Genes and Genomes (KEGG_website_https://www.genome.jp/kegg/), which was the novel data to study Canonical and non-canonical pathways.

Information of Database					Number of Gene Expression Changes	Gene Symbol	Fold Change
GSE10652	Hepatic ischemia	Liver	Mice	1-month 30/0 vs1-month Sham	7 genes changed (up_2, down_5)	NLRP12	−0.8883184
						**GBP7**	0.63616959
						**CASP4**	−0.5148444
						TXNIP	−0.408318
						**IFNAR2**	−0.3325283
						PLCB2	0.31739946
						TRPM7	−0.2500171
				1-month 60/0 vs 1-month Sham	12 genes changed (up_2, down_10)	**GBP7**	0.67488544
						CYBA	−0.4861952
						**CASP4**	−0.5421473
						CYBB	−0.9309736
						TXNIP	−0.4107721
						**IFNAR2**	−0.3163934
						GBP2	−0.2458004
						**JAK1**	−0.2762813
						TRPM7	−0.2834012
						CTSB	−0.2889644
						SUGT1	−0.3365294
						MAVS	0.3834712
						IL18	−0.2457362

				1-month 90/0 vs 1-month Sham	12 genes changed (up_1, down_11)	NLRP12	−0.8045909
						Naip1	−0.2710513
						ANTXR1	−0.2185604
						CYBA	−0.4167292
						CYBB	−0.8593563
						TXNIP	−0.5697741
						CTSB	−0.2697028
						**CASP4**	−0.4232423
						**JAK1**	−0.213004
						**GBP7**	0.62262234
						**IFNAR2**	−0.3502289
				12-months 30/0 vs 12 months Sham	4 genes changed (all downward trend)	CTSB	-0.2936041
						BRCC3	-0.3097179
						TRPM7	−0.3979388
						**JAK1**	−0.2981722
GSE10652	Hepatic ischemia	Liver	Mice	12-months 60/0 vs 12-months Sham	25 genes changed (up_2, down_23)	DNM1L	−0.7724573
						HSP90AA1	−1.2139601
						ANTXR2	−0.6353646
						HSP90AB1	−0.6384131
						BRCC3	−0.4454983
						PSTPIP1	0.4067073
						MFN2	−0.4102289
						SUGT1	−0.4848737
						IL18	−0.6284987
						**STAT1**	−0.3614548
						**IRF9**	−0.5036061
						NLRP3	0.3317576
						TRPM7	−0.4777352
						VDAC2	−0.5256487
						CTSB	−0.4755745

						CYBB	−0.8074354
						IFNAR1	−0.2711327
						NAMPT	−0.3410677
						CASP1	−0.3469305
						CASP8	−0.2745897
						MAVS	−0.3142809
						Naip5	−0.3532686
						NEK7	−0.2861765
						IFNAR2	−0.2513648
						Naip2	−0.5262612
				12−months 90/0 vs 12 months Sham	31 genes changes (up_1, down_30)	HSP90AA1	−1.3355584
						CTSB	−0.5847768
						SUGT1	−0.5610347
						DNM1L	−0.6721716
						HSP90AB1	−0.6883347
						GSDMD	−0.519367
						CYBB	−1.4804591
						BRCC3	−0.4619905
						STAT1	−0.5253528
						VDAC2	−0.4753058
						IL18	−0.5410453
						CYBA	−0.7075885
						IRF9	−0.5117166
						GBP3	−0.742158
						Naip2	−0.5942281
						PYCARD	−0.3001927
						MFN2	−0.2973127
						IFNAR2	−0.3707713
						NAMPT	−0.370094
						ANTXR2	−0.466616

						RIPK3	−0.2957112
						Naip5	−0.2982081
						PSTPIP1	0.2892862
						CASP8	−0.3945476
						**JAK1**	−0.4003779
						TRPM7	−0.4679364
						MAVS	−0.311644
						NEK7	−0.3016007
						RIPK1	−0.24208
						P2RX7	−0.2683878
						**IFNAR1**	−0.1850404

Table S2. 36 unique genes were significant changed (P value < 0.05 and Log2 fold change was shown in the last column) in young and adult mice of 90 minutes ischemia and 60 minutes reperfusion (I/R: 90/60) groups of the dataset GSE 10657. * Canonical genes (28) are shown in black and non-canonical genes (8) are shown in b old green.

Information of Database					Number of Gene Expression Changes	Gene Symbol	Fold Change (Log2FC)
GSE 10657	Hepatic ischemia and reperfusion	Liver	Mice	1-month 90/60 vs 1-month Sham	14 genes changed (up_3, down_11_Canonical_10 vs Non-canonical_4)	CYBB	−1.9928591
						NLRP12	−0.8727626
						CYBA	−0.9946454
						**JAK1**	−0.4738085
						IL1B	0.86277833
						GBP2	−0.3806487
						**GBP7**	0.66797064
						**IFNAR2**	−0.3811424
						CTSB	−0.615657
						BRCC3	−0.2585882
						**STAT2**	0.28543794
						GPRC6A	−0.292798
						SUGT1	−0.3563838

						RNASEL	−0.2121989
				12 months_ 90/60 vs 12 months Sham	31 genes changed (up_2, down_29_Canonical_25 vs Non-canonical_6)	CTSB	−0.750438
						CYBB	−1.8994705
						SUGT1	−0.5648423
						VDAC2	−0.5106923
						DNM1L	−0.7225227
						IL1B	0.67469313
						Naip2	−0.7188862
						IL18	−0.5313129
						**JAK1**	−0.6303857
						HSP90AB1	−0.5236145
						BRCC3	−0.4167281
						TRPM7	−0.5733089
						ANTXR2	−0.4131672
						Naip5	−0.4165443
						GSDMD	−0.4017083
						**IRF9**	−0.4864676
						**IFNAR2**	−0.3831382
						MFN2	−0.4182331
						**STAT1**	−0.4716966
						NAMPT	−0.3513346
						CYBA	−0.6368631
						NEK7	−0.3660494
						NLRP12	−0.5616419
						PSTPIP1	0.3570191
						**GBP3**	−0.4828349
						CASP8	−0.362241
						Naip1	−0.3331647
						TRPM2	−0.3689947
						ITPR1	−0.3045965

						P2RX7	−0.2435073
						IFNAR1	−0.1995226

Table S3. Summary table of 41 unique significantly downregulated (P value < 0.05) genes (Canonical_34 vs non-canonical_7) in young and adult mice after hepatic ischemia 90 minutes and reperfusion 0, and 60 minutes, respectively.

Symbol	Name	Inflammasome Stimuli	Inflammasome Component	Canonical vs Non-Canonical	Canonical vs Non-canonical Rationale
CYBB	Cytochrome b-245 beta chain (AKA GP91)	Hcys / Visfatin	NLRP3 Inflammasome	Canonical (82)	NLRP3 Inflammasome
CYBA	Cytochrome b-245 alpha chain (AKA GP91)	Hcys/Visfatin	NLRP3 Inflammasome	Canonical (82)	NLRP3 Inflammasome
CTSB	Cathepsin B	PAMP/DAMP Phagocytosis	NLRP3 Inflammasome	Canonical (82)	NLRP3 Inflammasome
NLRP12	NLR family pyrin domain containing 12 (Nlrp12)	Yersinia Bacteria	NLRP12 Inflammasome	Canonical (82)	NLRP12 Inflammasome
BRCC3	BRCA1/BRCA2-containing complex subunit 3	-	NLRP3 Inflammasome	Canonical (82)	Enhances NLRP3)
SUGT1	SGT1 homolog, MIS12 kinetochore complex assembly cochaperone(SUGT1)	-	NLRP3 Inflammasome	Canonical (82)	Enhances NLRP3)
IL18	Interleukin 18(Il18)	-	Canonical Pathway Component	Canonical (82)	Caspase-1 Cytokines Substrates
Naip1	NLR family apoptosis inhibitory protein 1(Naip1)	Gram-negative bacteria secretion system	NLRC4 Inflammasome	Canonical (82)	NLRC4 Inflammasome
HSP90AB1	Heat shock protein 90 alpha family class B member 1(HSP90AB1)	-	NLRP3 Inflammasome	Canonical (82)	Enhances NLRP3)
DNM1L	Dynamin 1 like	Viral RNA	NLRP3 Inflammasome	Canonical (82)	NLRP3 Inflammasome
P2RX7	Purinergic receptor P2X ligand-gated ion channel 7(P2rx7)	ATP/ K^+	NLRP3 Inflammasome	Canonical (82)	NLRP3 Inflammasome
VDAC2	Voltage dependent anion channel 2	Ca^{2+} Influx	NLRP3 Inflammasome	Canonical (82)	NLRP3 Inflammasome
GSDMD	Gasdermin D(Gsdmd)	-	Canonical Pathway Component	Canonical (82)	Non-canonical Inflammasome
MFN2	mitofusin 2	Viral RNA	NLRP3 Inflammasome	Canonical (82)	NLRP3 Inflammasome
Naip5	NLR family apoptosis inhibitory protein 5(Naip5)	Gram-negative bacteria secretion system	NLRC4 Inflammasome	Canonical (82)	NLRC4 Inflammasome
Naip2	NLR family apoptosis inhibitory protein 2(Naip2)	Gram-negative bacteria secretion system	NLRC4 Inflammasome	Canonical (82)	NLRC4 Inflammasome
ANTXR2	Anthrax toxin receptor 2	Bacterial Toxin	NLRP1 Inflammasome	Canonical (82)	NLRP1 Inflammasome
CASP8	Caspase 8(Casp8)	-	NLRP3 Inflammasome	Canonical (82)	Enhances Pro-CASP1)
NEK7	NIMA related kinase 7	-	NLRP3 Inflammasome	Canonical (82)	Enhances NLRP3)
NAMPT	Nicotinamide phosphoribosyl transferase (AKA VISFATIN)	Hcys/Visfatin	NLRP3 Inflammasome	Canonical (82)	NLRP3 Inflammasome
TRPM7	Transient receptor potential cation channel subfamily M member 7	Ca^{2+} Influx	NLRP3 Inflammasome	Canonical (82)	NLRP3 Inflammasome
ANTXR1	Anthrax toxin receptor 1	Bacterial Toxin	NLRP1 Inflammasome	Canonical (82)	NLRP1 Inflammasome
TXNIP	Thioredoxin interacting protein	Hcys / Visfatin	NLRP3 Inflammasome	Canonical (82)	NLRP3 Inflammasome
GPRC6A	G protein-coupled receptor class C group 6 member A	Ca^{2+} Influx	NLRP3 Inflammasome	Canonical (82)	NLRP3 Inflammasome
GBP2	Guanylate binding protein 2	Viral DNA/ Cytosolic Bacteria	AIM2 Inflammasome	Canonical (82)	AIM2 Inflammasome
RNASEL	Ribonuclease L	Viral RNA	NLRP3 Inflammasome	Canonical (82)	NLRP3 Inflammasome
Symbol	Name	Inflammasome Stimuli	Inflammasome Component	Canonical vs Non-Canonical	Canonical vs Non-canonical Rationale
MAVS	Mitochondrial antiviral signaling protein	Viral RNA	NLRP3 Inflammasome	Canonical (82)	NLRP3 Inflammasome

RIPK1	Receptor interacting serine/threonine kinase 1	Viral RNA	NLRP3 Inflammasome	Canonical (82)	NLRP3 Inflammasome
HSP90AA1	Heat shock protein 90 alpha family class A member 1(HSP90AA1)	-	NLRP3 Inflammasome	Canonical (82)	Enhances NLRP3
RIPK3	Receptor interacting serine/threonine kinase 3	Viral RNA	NLRP3 Inflammasome	Canonical (82)	NLRP3 Inflammasome
PYCARD	PYD and CARD domain containing(PYCARD)	-	Canonical Pathway Component	Canonical (82)	Inflammasome Components
IL1B	Interleukin 1 beta(Il1b)	-	Canonical Pathway Component	Canonical (82)	Caspase 1 Cytokines Substrates
TRPM2	Transient receptor potential cation channel subfamily M member 2	Ca^{2+} Influx	NLRP3 Inflammasome	Canonical (82)	NLRP3 Inflammasome
ITPR1	Inositol 1,4,5-trisphosphate receptor type 1	Ca^{2+} Influx	NLRP3 Inflammasome	Canonical (82)	NLRP3 Inflammasome
IFNAR2	Interferon alpha and beta receptor subunit 2	Non-canonical Pathway Stimuli	Non-canonical Inflammasome	Non-canonical (14)	Non-canonical Inflammasome
JAK1	Janus kinase 1	Non-canonical Pathway Stimuli	Non-canonical Inflammasome	Non-canonical (14)	Non-canonical Inflammasome
GBP3	Guanylate binding protein 3	Non-canonical Pathway Stimuli	Non-canonical Inflammasome	Non-canonical (14)	Non-canonical Inflammasome
IRF9	Interferon regulatory factor 9	Non-canonical Pathway Stimuli	Non-canonical Inflammasome	Non-canonical (14)	Non-canonical Inflammasome
STAT1	Signal transducer and activator of transcription 1	Non-canonical Pathway Stimuli	Non-canonical Inflammasome	Non-canonical (14)	Non-canonical Inflammasome

IFNAR1	Interferon alpha and beta receptor subunit 1	Non-canonical Pathway Stimuli	Non-canonical Inflammasome	Non-canonical (14)	Non-canonical Inflammasome
CASP4	Caspase 4(CASP4)	Non-canonical Pathway Stimuli	Non-canonical Inflammasome	Non-canonical (14)	Non-canonical Inflammasome

Table S4. Summary table of seven unique significantly upregulated (P value < 0.05) genes (Canonical_5 vs non-canonical_2) in young and adult mice after hepatic ischemia 90 minutes and reperfusion with 0, and 60 minutes, respectively.

Symbol	Name	Inflammasome Stimuli	Inflammasome Component	Canonical vs Non-Canonical	Canonical vs Non-canonical Rationale
GBP7	Guanylate binding protein 7	Non-canonical Pathway Stimuli	Non-canonical Inflammasome	Non-canonical (14)	Non-canonical Inflammasome
PLCB2	Phospholipase C beta 2	Ca^{2+} Influx	NLRP3 Inflammasome	Canonical (82)	NLRP3 Inflammasome
MAVS	Mitochondrial antiviral signaling protein	Viral RNA	NLRP3 Inflammasome	Canonical (82)	NLRP3 Inflammasome
PSTPIP1	Proline-serine-threonine phosphatase interacting protein 1(PSTPIP1)	-	NLRP3 Inflammasome	Canonical (82)	Inhibits ASC
NLRP3	NLR family pyrin domain containing 3(NLRP3)	-	NLRP3 Inflammasome	Canonical (82)	NLRP3 Inflammasome
IL1B	Interleukin 1 beta(Il1b)	-	Cannonical Pathway Component	Canonical (82)	Caspase 1 Cytokines Substrates
STAT2	Signal transducer and activator of transcription 2	Non-canonical Pathway Stimuli	Non-canonical Inflammasome	Non-canonical (14)	Non-canonical Inflammasome

Table S5. 45 unique pyroptosis genes were significant changed (P value < 0.05 and fold change (Log2FC) was shown in the last column) in 48 male rats that were subjected to liver ischemia-reperfusion (IRI), ischemic pre- (IPC), post-conditioning (IPO), and IPC+IPO in the dataset of GSE24430.

Information of Dataset		Organ	Model	Comparison group	Number of gene expression changes	Gene Symbol	Fold Change(log2FC)
GSE24430	The effects of ischemic Pre- and Post Conditioning on rat liver after Ischemia/Reperfusion	Liver	Male Rat	IRI(n=10) vs Sham (n=8)	6 genes changed (up_2, down_4_All Canonical genes)	CASP1	−0.3462926
						VDAC3	0.4137129
						ITPR1	−0.4229094
						TXN2	0.4461861
						TXNIP	−0.4138986
						DNM1L	−0.3817125
				IPC (n=10) vs Sham (n=8)	18 genes changed (up_16, down_2_Canonical_16 vs Non-canonical_2)	GBP2	0.2569839
						GBP5	0.3271734
						GSDMD	0.3340227
						Naip6	−0.3235036
						PRKCD	0.3082567
						TRPM2	0.3347097
						VDAC2	0.3806432
						VDAC3	0.6501832
						ITPR1	−0.3499832
						MCU	0.4405819
						CASP12	0.9016174
						CYBB	0.40808
						NAMPT	1.0139753
						CTSB	0.3555571
						RIPK1	0.3192147
						JAK1	0.4922222

						STAT1	0.3154483
						RHOA	0.2400502

Information of Dataset		Organ	Model	Comparison group	Number of gene expression changes	Gene Symbol	Fold Change (log2FC)
GSE24430	The effects of ischemic Pre- and Post Conditioning on rat liver after Ischemia/Reperfusion	Liver	Male Rat	IPO (n=10) vs Sham (n=8)	39 genes changed (up_32, down_7_Canonical_33 vs Non-canonical_6)	AIM2	−0.2577251
						GBP5	0.31822
						IL18	0.2962696
						PYCARD	0.3786387
						GSDMD	0.4575344
						Naip6	−0.236961
						PRKCD	0.3709584
						ANTXR1	−0.2923983
						NOD2	−0.2272295
						TRPM2	0.3919706
						TRPM7	0.3723366
						TRPV2	−0.2250771
						VDAC1	0.5804169
						VDAC2	0.5600052
						VDAC3	0.8049723
						GPRC6A	−0.2865448
						ITPR1	−0.3923547
						MCU	0.5805616

						PLCB3	0.1728649
						CASP12	1.1612324
						HSP90AA1	0.7356158
						HSP90AB1	0.360229
						SUGT1	0.6002512
						NEK7	0.3473913
						FADD	0.310122
						TXN2	0.3266655
						CTSB	0.3735941
						DHX33	0.356758
						MAVS	0.3436675
						MFN1	0.3731446
						MFN2	0.3598689
						RIPK1	0.4698851
						CASP4	0.4989304
						JAK1	0.5392736
						STAT1	0.3350231
						STAT2	0.4114075
						IFNAR1	0.2804942
						IRF9	0.5742993
						RHOA	0.3528287
				IPC+IPO (n=10) vs Sham (n=8)	14 genes changed (up_13, down_1_Canonical_11 vs Non-canonical_3)	Naip6	−0.2124224
						PRKCD	0.29353805
						VDAC1	0.37337835
						VDAC2	0.514385
						VDAC3	0.7136301
						MCU	0.5020594

						CASP12	0.62123152
						HSP90AA1	0.35937495
						SUGT1	0.45029757
						NAMPT	0.68208562
						MAVS	0.27835283
						JAK1	0.4652942
						IFNAR1	0.3293964
						IRF9	0.4130613

Table S6. 49 unique trained immunity pathway enzymes were significant changed (P value < 0.05 and fold change (Log2FC) was shown in the last column) in 48 male rats that were subjected to liver ischemia-reperfusion (IRI), ischemic pre- (IPC), post-conditioning (IPO), and IPC+IPO in the dataset of GSE24430.

Dataset	Information of Dataset	Classification of trained immunity gene group	Number of gene expression changes	Gene Symbol	Fold Change (Log2FC)
**GSE 24430**	Group of IRI	Glycolysis	6 genes changed (up_3, down_3)	AKR1A1	0.6189636
				ALDH1A3	−0.2421692
				GCK	0.5071392
				PFKFB1	−0.8155308
				PFKM	−0.1880415
				TPI1	0.5701765
		Mevalonate pathway	3 genes changed (up_3, down_0)	MVK	0.4054709
				MVD	0.4974663
				PMVK	0.8406006
		Acetyl-CoA generation enzyme	No significant changed genes		
		Glycolysis		ACSS2	0.4848061

	Group of IPC		26 genes changed (up_24, down_2)	ADH7	0.5049168
				AKR1A1	0.8594253
				ALDH1A3	-0.2310607
				ALDH1B1	0.8172076
				ALDH2	0.390866
				ALDH3A2	0.4404758
				ALDOA	0.3948963
				ENO1	0.6632549
				FBP1	0.493132
				G6PC	0.543041
				GAPDH	0.4939365
				GCK	1.1820065
				PANK1	0.7632486
				PCK1	0.542632
				PDHA1	0.5848095
				PDHB	0.4360867
				PFKFB1	−1.0439883
				PFKFB3	0.3801202
				PFKL	0.5814004
				PGAM1	0.4117774
				PGK1	0.4936424
				PGM1	0.7633234
				PKLR	0.8958553
				PKM	0.1967362
				TPI1	0.561594
		Acetyl-CoA generation enzyme	9 genes changed (up_9, down_0)	ALDH2	0.390866
				HADH	0.3915323
				ACAA2	0.3285622
				BDH1	0.2862645
				IDH1	0.4346336
				ACLY	0.7295146
				ACSS2	0.4848061
				ACLY	0.7295146

				ACSS2	0.4848061
		Mevalonate pathway	4 genes changed (up_9, down_0)	HMGCS1	0.6987006
				MVK	0.5415997
				MVD	0.521872
				PMVK	1.0522692
	Group of IPO	Glycolysis	37 genes changed (up_29, down_8)	ACSS2	0.4317393
	Group of IPO **Group of IPC+IPO**	Glycolysis	37 genes changed (up_29, down_8)	ADH7	0.4822883
				AKR1A1	0.9172192
				ALDH1A3	-0.256197
				ALDH1B1	0.7274625
				ALDH2	0.508768
				ALDH3A2	0.4277597
				ALDH9A1	0.4432192
				ALDOA	0.2363083
				ALDOB	0.2673237
				DLAT	0.4449253
				DLD	0.556987
				ENO1	0.8104452
				FBP1	0.510858
				G6PC	0.737604
				GAPDH	0.5747515
				GAPDHS	-0.3639551
				GCK	0.7897331
				GPI	0.3009449
				HK2	−0.3153311
				LDHAL6B	−0.2250985
				LDHC	−0.2102048
				PANK1	0.7609183
				PCK1	0.4453

				PDHA1	0.6140545
				PDHB	0.5092503
				PFKFB1	−0.5642344
				PFKFB4	−0.2738591
				PFKL	0.6403217
				PGAM1	0.6463801
				PGK1	0.621269
				PGK2	−0.3884691
				PGM1	0.909837
				PKLR	0.9971398
				PKM	0.3398105
				SLC2A2	0.3640554
				TPI1	0.6195364
				ALDH2	0.508768
		Acetyl-CoA generation enzyme	9 genes changed (up_9, down_0)	HADH	0.4455692
				ACAA2	0.4376583
				BDH1	0.4206116
				IDH1	0.6205776
				ACLY	0.8698953
				ACSS2	0.4317393
				ACLY	0.8698953
				ACSS2	0.4317393
				HMGCS1	0.5915918
		Mevalonate pathway	4 genes changed (up_4, down_0)	MVK	0.4745986
				MVD	0.2419685
				PMVK	1.1022705
**GSE 24430**	**Group of IPC+IPO**	**Glycolysis**	**19 genes changed (up_18, down_1)**	AKR1A1	0.77502845
				ALDH1B1	0.56416063
				ALDH2	0.391855

				ALDH3A2	0.4174824
				BPGM	0.39403415
				ENO1	0.49030493
				FBP1	0.423226
				GAPDH	0.378576
				GCK	0.7847054
				PANK1	0.68562635
				PDHA1	0.5189199
				PFKFB1	−0.5563169
				PFKFB3	0.3772673
				PFKL	0.49417472
				PGAM1	0.46907
				PGK1	0.4897786
				PGM1	0.69454172
				TPI1	0.5368874
				**ADH7**	**0.27274123**
		Acetyl-CoA generation enzyme	4 genes changed (up_4, down_0)	HADH	0.43041225
				ACAA2	0.39765825
				IDH1	0.47190162
				ALDH2	0.391855
		Mevalonate pathway	2 genes changed (up_2, down_0)	MVK	0.452409
				PMVK	0.8019878
